# Targeted cleavage of *nad6* mRNA induced by a modified pentatricopeptide repeat protein in plant mitochondria

**DOI:** 10.1038/s42003-018-0166-8

**Published:** 2018-10-11

**Authors:** Catherine Colas des Francs-Small, Lilian Vincis Pereira Sanglard, Ian Small

**Affiliations:** Australian Research Council Centre of Excellence in Plant Energy Biology, School of Molecular Sciences, The University of Western Australia, Crawley, WA 6009 Australia

## Abstract

Mitochondrial genes encode key components of the cellular energy machinery, but their genetic analysis is difficult or impossible in most organisms (including plants) because of the lack of viable transformation approaches. We report here a method to block the expression of the mitochondrial *nad6* gene encoding a subunit of respiratory complex I in *Arabidopsis thaliana*, via the modification of the specificity of the RNA-binding protein RNA PROCESSING FACTOR 2 (RPF2). We show that the modified RPF2 binds and specifically induces cleavage of *nad6* RNA, almost eliminating expression of the Nad6 protein and consequently complex I accumulation and activity. To our knowledge, this is the first example of a targeted block in expression of a specific mitochondrial transcript by a custom-designed RNA-binding protein. This opens the path to reverse genetics studies on mitochondrial gene functions and leads to potential applications in agriculture.

## Abstract (web summary)

Catherine Colas des Francs-Small et al. used an engineered pentatricopeptide repeat protein to induce cleavage of *nad6* mRNA in the mitochondria of *Arabidopsis thaliana*, eliminating its expression. The approach has potential for use in functional characterization of mitochondrial genes and future agricultural applications.

## Introduction

Among the few genetic mutations known in plant mitochondria^1^ are recombinant open reading frames (ORFs) linked to cytoplasmic male sterility (CMS)^2,3^. CMS is widely used in agriculture to facilitate the production of hybrid lines^3^. CMS can be suppressed by restorer-of-fertility genes, many of which encode pentatricopeptide repeat (PPR) proteins^4,5^. PPR proteins are sequence-specific RNA-binding proteins involved in posttranscriptional stages of organelle transcript maturation^6^. Their modular and predictable interactions with RNA make them amenable to custom modification and design^7,8^. Angiosperms contain about 500 different PPR separable into two classes (P-class and PLS-class) distinguished by their characteristic patterns of repeated motifs^9^. Restorer-of-fertility proteins form a clade within the P-class PPR proteins that co-evolves with mitochondrial ORFs causing CMS^4^. They appear to block expression of specific mitochondrial ORFs by inducing RNA cleavage or degradation, or by preventing translation. The mechanisms by which these processes are achieved are not known in detail^5,10^. *Arabidopsis thaliana* has 26 restorer-of-fertility-like (RFL) proteins^11^, some of which are involved in 5′-end processing of mitochondrial transcripts^12–14^.

The discovery of the PPR code^15^ describing the correspondence between key residues of PPR motifs and each base of its target RNA^8^ allows the prediction of a target for a given PPR protein^16,17^, or inversely the prediction of which PPR protein binds a given target^18^. It also permits the design of proteins with altered binding capabilities^8,15,19,20^, suggesting that custom RNA-processing factors with desired targeting specificities could be developed^7^. In this work, we designed the RFL protein RNA PROCESSING FACTOR 2 (RPF2)^13^ to bind a new RNA target, located within the coding sequence of *nad6*, and show that the re-targeting is successful.

## Results

### Re-design and expression of RPF2

**Fig. 1:**
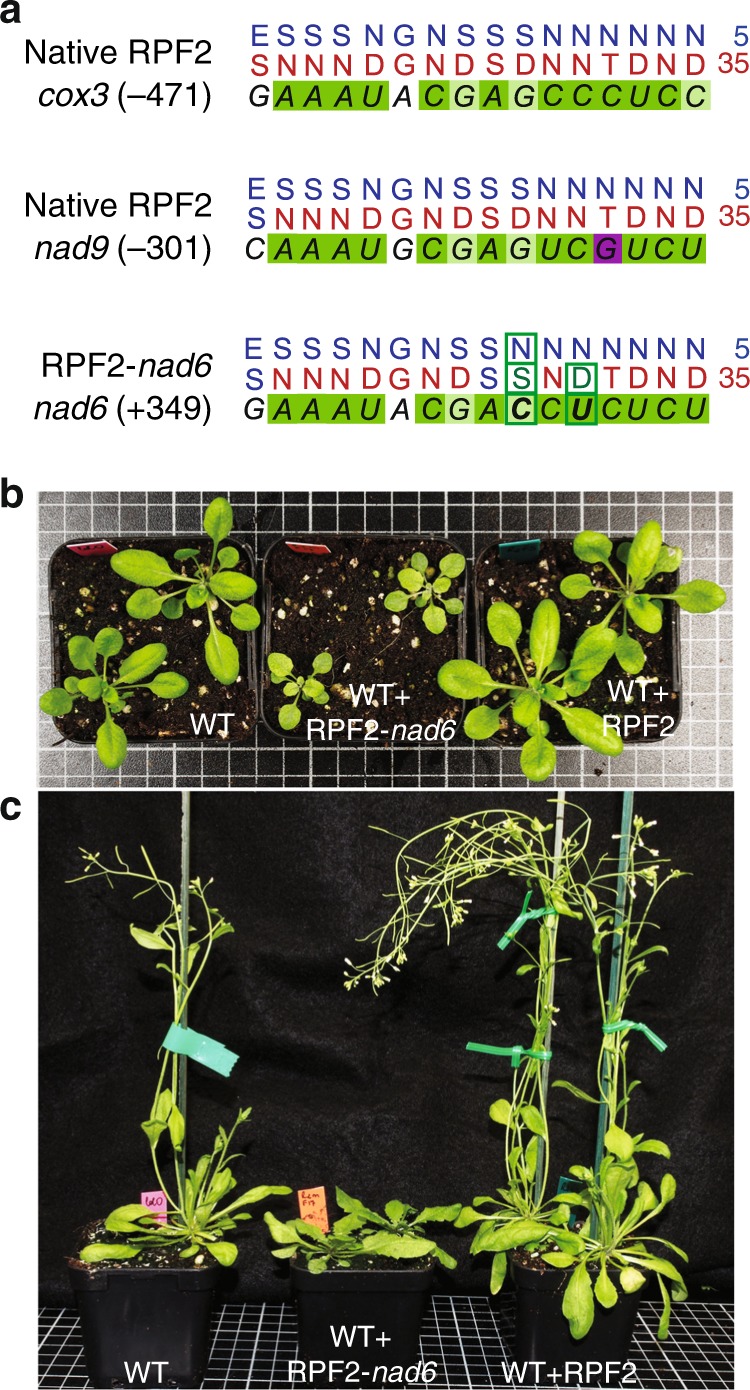
Targeting *nad6* with a modified RPF2 protein leads to plants with a slow growth phenotype. **a** Binding predictions for the RPF2 and RPF2-*nad6* proteins on their respective targets (5′-UTRs of *cox3* and *nad9* for RPF2 and the coding sequence of *nad6* for RPF2-*nad6*). Dark green represents a perfect match, light green a partial match, and magenta a mismatch according to the PPR code^15^. **b**, **c** Phenotypes of plants transformed with native RPF2 or RPF2-*nad6* compared with WT. **b** Four-week-old rosette; **c** 6-week-old plants grown under 18 h photoperiod

RPF2 (At1g62670) has 16 PPR motifs and 2 known natural targets, located within the 5′-untranslated regions (UTRs) of *cox3* and *nad9*^13^. Using the code defined by Barkan et al.^15^, RPF2 is predicted to bind to similar sequences 471–455 nt upstream of the *cox3* start codon and 301–286 nt upstream of the *nad9* start codon (Fig. 1a)^21^. We searched for other similar sequences in the *A. thaliana* mitochondrial transcriptome, and found that the sequence at position + 349–365 in the coding sequence of *nad6* has only 3 differences to the predicted RPF2-binding site in the *cox3* transcript. We modified the coding sequence of RPF2 to incorporate residues that would be predicted to recognize this new target, giving the sequence that we refer to as RPF2-*nad6* (Supplementary Fig. 1). RPF2-*nad6* was introduced into *A. thaliana* Col-0 plants via *Agrobacterium* infection. A similar construct with the native RPF2 cDNA was transformed in parallel as a control. Reverse-transcriptase PCR (RT-PCR) was performed on primary transformants (T1) to ensure that the constructs were integrated in the genome and expressed (Supplementary Fig. 2). The FLAG-tagged protein could not be detected by western blotting in mitochondrial protein extracts, but 12 out of 19 independent transformants carrying the RPF2-*nad6* construct displayed slow growth (Fig. 1b), and later in development, dark curled foliage reminiscent of *Arabidopsis* complex I mutants (Fig. 1c and Supplementary Fig. 3). All control plants (wild type (WT) + native RPF2) were phenotypically indistinguishable from WT (23 independent transformants).

### RPF2-*nad6* plants have reduced Nad6 and complex I

**Fig. 2:**
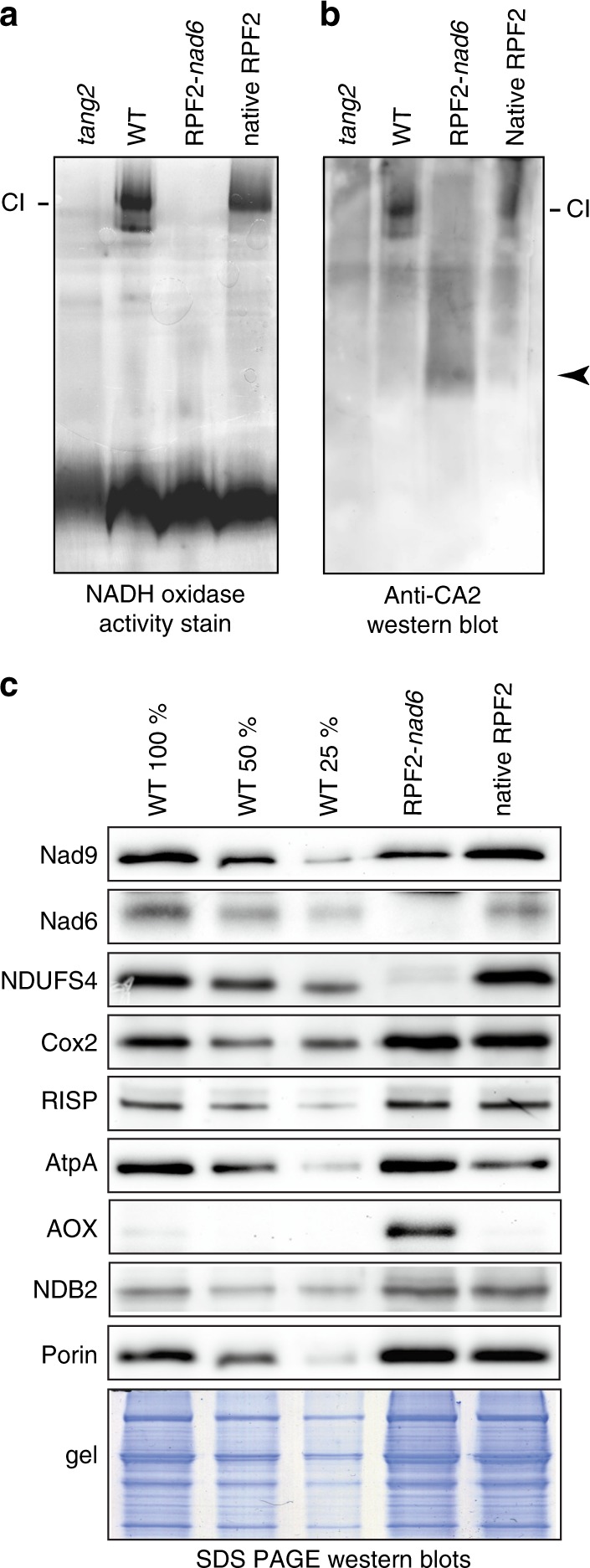
RPF2-*nad6* plants lack assembled respiratory complex I. **a**, **b** Separation of mitochondrial inner membrane protein complexes by blue native PAGE in RPF2-*nad6* plants as compared with WT, *tang2* (a complex I mutant) and plants transformed with the native RPF2 constructs (RPF2). NADH oxidase activity was revealed in-gel (**a**), showing the lack of assembled complex I in the RPF2-*nad6* plants. A western blotting of a similar gel was probed with an anti-CA2 antibody (**b**). The black arrow shows a low-molecular-weight assembly intermediate of complex I in the RPF2-*nad6* plants. **c** Western blottings of mitochondrial proteins from RPF2-*nad6* plants as compared with WT and control RPF2 plants separated by SDS-PAGE. Uncropped images of the blots are presented in Supplementary Fig. 4

In order to check whether the phenotypes observed could be linked to an alteration in complex I levels, blue native polyacrylamide gel electrophoresis (BN-PAGE) was performed on isolated leaf mitochondria from T2 plants of the transformants (Fig. 2a). No NADH oxidase activity from complex I was revealed in mitochondria from the slow growing plants bearing the RPF2-*nad6* construct. Western blotting of the BN-PAGE gel and probing with an antibody raised against the carbonic anhydrase subunit CA2, one of the first subunits to be incorporated in complex I^22^, revealed assembled complex I in the WT mitochondria and the RPF2 plants (Fig. 2b) but only a low-molecular-weight subcomplex in RPF2-*nad6* mitochondria, suggesting that the membrane arm assembly and subsequent complex assembly were compromised in the latter. This low-molecular-weight sub-complex was not detected in *tang2*, a PPR mutant altered in the splicing of the *nad5* transcript^23^. These results are consistent with a proposed order of assembly of complex I subunits in which CA2 and Nad2 are involved in the assembly of an initial 200 kDa subcomplex, with Nad6 addition required for the next stage of assembly^22,24–26^. Further analysis of respiratory complex subunits by SDS-PAGE and western blotting (Fig. 2c, Supplementary Fig. 4) showed that Cox2 (complex IV), RISP (complex III), and AtpA (complex V) subunits were unchanged in RPF2-*nad6* mitochondria, but Nad9 was decreased and Nad6 was undetectable in mitochondria of RPF2-*nad6*-transformed plants (Fig. 2c). Very low levels of NDUFS4, a nuclearly encoded subunit of complex I, were detected. Furthermore, alternative oxidase (AOX) was greatly induced in RPF2-*nad6* plants (Fig. 2c), as commonly observed for mutants with altered complex I function^27–29^.

### The *nad6* transcript is cleaved in RPF2-*nad6* plants

**Fig. 3:**
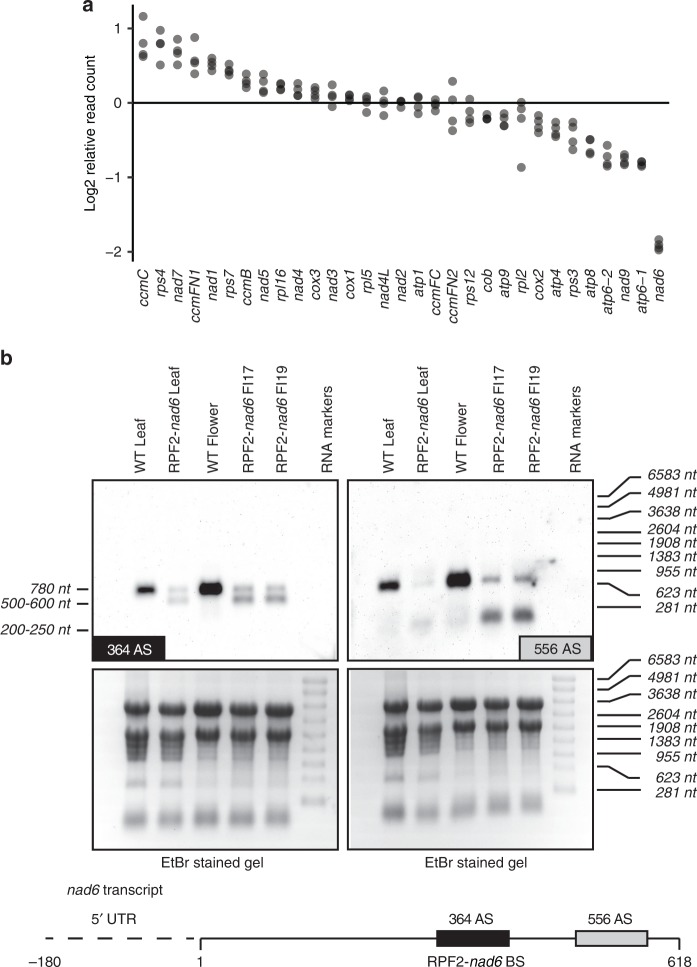
The *nad6* transcript is cleaved in the plants transformed with the RPF2-*nad6* constructs. **a** The plot shows the relative expression of all mitochondrial transcripts in RPF2-*nad6* plants compared with WT as log2 ratios of read counts. Each point represents one of four biological replicates. The reduced expression of *nad6* in RPF2-*nad6* plants is significant (FDR-corrected *p*-value 6.3 × 10^−78^; Wald’s test using DESeq2^42^). **b** Northern blottings of leaf and flower RNAs from two independent RPF2-*nad6* transformants and WT plants (top panels). The probe 364 AS covers the predicted RPF2-*nad6*-binding site and 556 AS hybridizes 3′ of the binding site. The lower panels show the gels stained with ethidium bromide before transfer to control for loading differences. The sizes on the left indicate estimated sizes of the hybridizing RNAs, sizes on the right are markers

A quantitative analysis of the expression levels of mitochondrial transcripts in the modified RPF2 plants as compared with WT was obtained by RNA sequencing (RNA-seq). This analysis revealed that levels of the *nad6* transcript were reduced about fourfold in RPF2-*nad6* (Fig. 3a). An explanation for this reduction was sought through northern blotting with *nad6*-specific probes. Leaf and flower RNAs were probed with biotinylated oligonucleotide probes targeted to the vicinity of the predicted RPF2-*nad6* binding site (Fig. 3b). Probe 364 AS (covering the predicted binding site) hybridized to a single ~780 nt transcript in WT samples corresponding to the expected size of the mature *nad6* transcript (179 nt 5′-UTR followed by 601 nt of coding sequence—the *nad6* mRNA in *A. thaliana* has no stop codon or 3′-UTR^30^). In the RPF2-*nad6* plants, this hybridization signal was much weaker and accompanied by a more intense signal migrating at ~500–600 nt, suggesting that the *nad6* transcript is cleaved in the RPF2-*nad6* plants. With the 556 AS probe (hybridizing 3′ of the predicted binding site), a second potential cleavage product was detected, migrating at ~200–250 nt.

**Fig. 4:**
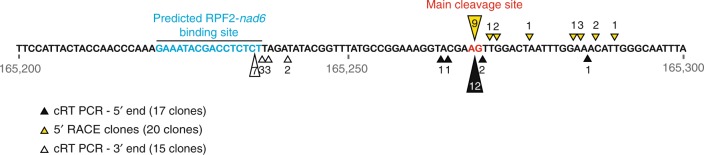
Recapitulative map of *nad6* transcript cleavage in RPF2-*nad6* plants using the 5′-RACE and cRT-PCR results. The coordinates of this region on the Col-0 mitochondrial genome are 165,200 to 165,300. The predicted RPF2-*nad6* binding (165,221–165,236) and cleavage (165,269) sites are highlighted. The black and yellow triangles indicate the 5′-ends of the cleaved products as determined by cRT-PCR and 5′-RACE PCR, respectively. White triangles show the 3′-ends of the cleaved products as determined by cRT-PCR. The figures near the triangles indicate the numbers of clones obtained

To map the exact site of the apparent cleavage, we performed 5′-rapid amplification of cDNA end (RACE) PCR and circular RT-PCR (cRT-PCR) experiments. The sizes of the 5′-RACE and cRT-PCR products were determined by migration on a 3% low melting agarose gel and sequencing of cloned products (Supplementary Fig. 5). Multiple transcript ends could be mapped within a region encompassing 55 nucleotides beginning at or near the end of the predicted RPF2-*nad6*-binding site (Fig. 4). The cRT-PCR revealed that the cleaved RNA is lacking a segment of ~33 nt. This could result either from a double endonucleolytic cleavage or a single cleavage followed by exonucleolytic trimming (Supplementary Fig. 6). We found no evidence for the additional product expected from a double cleavage using RACE with primers targeted within this segment, or by sequencing small (–17-50 nt) RNAs prepared from WT and RPF2-*nad6* plants (Supplementary Fig. 7). The 5′–3′ exonuclease activity is unknown from plant mitochondria^31^; thus, we suggest that the initial cleavage is 55 nt 3′ of the RFP-*nad6*-binding site, followed by 3′–5′ exonucleolytic degradation, which is inhibited from extending further by the bound PPR protein. This scenario is supported by the observation of un-templated A addition at the 3′-end of the 5′-cleavage product (Supplementary Fig. 5), known to induce 3′–5′ exonuclease activity by polynucleotide phosphorylase^32^.

### Off-target cleavage of *rps3-rpl16* transcripts

The cleavage of *nad6* transcripts in close proximity to the predicted RPF2-*nad6* binding site and the mapping of an RNA end at this site strongly suggests that RPF2-*nad6* does indeed recognize the target sequence it was designed to bind. Although the RNA-seq data (Fig. 3a) did not provide obvious evidence of loss of any other transcripts besides *nad6*, this does not rule out more subtle off-target effects. We searched for other potential targets in the mitochondrial transcriptome with the pattern ‘NAAAURCGACCUNUCY’ (based on the *nad6* target site, but allowing redundancy where the natural targets of RPF2 suggest base-specificity is less than perfect). A single sequence matches exactly (the *nad6* site), but a sequence in the *rps3* coding sequence contains only one minor (C–U) mismatch to this pattern, and as distinction between C and U appears to be the most difficult for PPR motifs^19^, we investigated potential cleavages in the vicinity of this site (Supplementary Fig. 8). We discovered that the *rps3-rpl16* co-transcript is cleaved ~20 nt 3′ of the predicted binding site, leading to accumulation of the 3′ end of the transcript but loss of the 5′-part; expression of Rps3 and Rpl16 is presumably unaffected because the full-length transcript containing both overlapping reading frames is present at WT levels in the RPF2-*nad6* plants. Thus, as seen for natural RPF2, RPF2-*nad6* can tolerate mismatches to its predicted preferred target sequence, leading to ‘off-target’ RNA cleavage.

### The endonuclease that cleaves remains unidentified

An interesting feature of the cleavages induced by RPF2-*nad6* is their distance from the predicted binding sites. This “cleavage at a distance” is also inferred to occur with the RNAs that are natural targets of unmodified RPF2. The predicted binding sites on *nad9* and *cox3* are, respectively, 83 nt and 77 nt 5′ of the final mRNA termini in Col-0 (Supplementary Fig. 9). Thus, identical (or near-identical) proteins apparently induce cleavages at different distances on different RNAs. What might be the mechanism? The relatively long distances to the proposed cleavage sites suggest the involvement of another factor, and/or RNA secondary structure. The tRNA processing enzyme proteinaceous RNAse P (PRORP)^33^ was implicated in the RFL2-promoted cleavage of *orf291*^10^ and is proposed to cleave *nad6* mRNA 17 nt upstream of its stop codon^34^. In both cases, tRNA-like secondary structures can form that are a substrate for PRORP^35^. We could find no such structure in the vicinity of the RPF2-*nad6* cleavage sites or near the RPF2 cleavage sites in *nad9* and *cox3* (Supplementary Fig. 10), making it unlikely that PRORP is the endonuclease in these cases. We also conclude from these folding simulations that binding of RPF2 or RPF2-*nad6* does not obviously alter the structure of the RNA around the cleavage sites. The endonucleases MNU1 and MNU2 have been proposed to act in concert with RPF2 to cleave *nad9* and *cox3*^36^, but we still observed RPF2-*nad6*-induced cleavage of *nad6* in a double *mnu1 mnu2* mutant background (Supplementary Fig. 11, 12). The identity of the endonuclease presumed to be working with RFP2-*nad6* is thus still obscure.

## Discussion

We have shown that it is possible to redirect an RFL-type PPR protein to a new target within mitochondria in vivo and effectively block expression of the target transcript via induced cleavage of the mRNA and a resulting loss of the translation product. To our knowledge, it is the first time a modified PPR protein has been used to block the expression of an organellar transcript in this way. A previous attempt to achieve a similar result, the directed knockdown of *matR*, the mitochondrial maturase whose gene lies within *nad1* intron 4, used synthetic ribozymes^37^. The knockdown effect yielded by this technique was at best a 50% reduction in the transcript and protein, much less than the fourfold decrease in transcript levels and almost total lack of Nad6 protein achieved in this work. We believe that the approach we demonstrate here should provide a general means to implement reverse genetics approaches in plant mitochondria given the ease with which PPR proteins can be designed to recognize different target sequences^7,20^. Extension of this approach to plastids is possible in principle, but will probably require the addition of an endonuclease domain to the PPR; there is no evidence so far that RFL proteins can induce cleavage of plastid RNAs. Lack of specificity (particularly distinction between C and U) remains an issue to be solved. One possible approach may be to reduce the length of the PPR tract; experiments with synthetic PPR proteins suggest that 10 PPR motifs are sufficient for optimal binding, whereas 14 motifs allow the protein-RNA complex to tolerate more mismatches^19^. Natural PPR proteins are often longer than this and can bind a range of similar sequences, as demonstrated for the maize plastid protein PPR10^38^. Natural RFL proteins contain up to 15–20 PPR motifs^4,11^, implying a similar ability to bind a range of related targets, in accordance with the observed ability of RPF2 and RPF2-*nad6* (16 motifs) to bind two closely related sequences each.

## Methods

### Cloning

The synthetic RPF2-*nad6* gene as well as a fragment containing the NOS promoter, the 25 amino acid *Solanum tuberosum* formate dehydrogenase (FDH) targeting peptide and a FLAG tag were commercially synthesized, digested with BspHI and BamHI, and EcoRI and BspHI, respectively, and cloned together into the pCAMBIA1380 binary vector^39^. The native RPF2 gene was amplified from Col-0 genomic DNA with the Takara PrimeSTAR HS polymerase using the primers RPF2pET*Rca*F and RPF2pET*Bam*H1R (Supplementary Table 1) and cloned with the same promoter-targeting sequence and FLAG cassette in pCAMBIA1380 (Supplementary Fig. 1a). The synthetic genes were transferred to *Agrobacterium tumefaciens* and introduced into *A. thaliana* plants by floral dip^40^. In short, floral stems carrying unpollinated buds were dipped for 20–30 s into a fresh culture of *A. tumefaciens* resuspended in a 10% sucrose solution containing 0.5 μL mL^−1^ Silwett. The plants were covered and kept under low light for 24 h, staked, and grown until the siliques were ready to harvest. The transformants were selected on 25 μg mL^−1^ hygromycin.

### RNA sequencing

Total RNA was isolated from 6-week-old rosette leaves and bolting flower buds of Col-0 and one of the RPF2-*nad6* transformed lines (T2) with an RNeasy Qiagen kit. Four independent libraries for each genotype were made from 500 ng of Turbo DNase (Ambion) treated total RNA using an Illumina TruSeq Stranded kit. The sequencing run (MiSeq Reagent Kit v3, 150 cycles) was performed on an Illumina MiSeq sequencer. Reads were mapped to the Col-0 mitochondrial transcriptome (derived from accession JF729201) with Salmon^41^ v0.11.2 and the count data analyzed with DESeq2^42^ v1.20.0.

### Northern blotting

RNA from rosette leaves and flowers was extracted using PureZol (Bio-Rad). Eight micrograms of total RNA were run on a 1.2 % denaturing agarose gel and transferred onto Hybond N + membrane (Amersham). Northern blotting was performed as described previously^43^ using oligonucleotide probes labeled in 5′ with biotin (Supplementary Table 1). The membranes were pre-hybridized for 1–2 h at 50 °C in 5 × SSC, 7% SDS, 100 μg ml^−1^ heparin, 20 mM Na_2_HPO_4_ pH 7.5 and hybridized overnight in the same buffer containing 1 nM biotinylated probe. Three short washes in 3 × SSC, 5% SDS, 25 mM Na_2_HPO_4_ pH 7.5 were performed at room temperature. The northern blottings were developed with the Pierce Chemiluminescent Nucleic Acid Detection Module Kit.

### 5′-RACE PCR

5′-RACE was performed using the SMARTer RACE cDNA Amplification Kit (Clontech) according to the manufacturer’s instructions. Random hexamers were used to prime reverse transcription. The PCR amplification was done using a specific primer in the *nad6* coding sequence (*nad6*-554Rv, Supplementary Table 1). The RACE PCR products obtained for the Col-0 and RPF2-*nad6* plants were run on a 3% low melting agarose gel (Supplementary Fig. 5), cloned into pGEM-T Easy (Promega) and sequenced.

### Circular RT-PCR

Crude preparations of mitochondria were isolated from 4-week-old Col-0 and RPF2-*nad6* seedlings grown on half-strength Murashige and Skoog medium. Mitochondrial RNA was purified from mitochondrial pellets using PureZol (Bio-Rad) and for each reaction, 2–3 μg were treated with Turbo DNase (Ambion). RNA was circularized using the T4 RNA ligase and reverse transcription was performed with the Superscript III (Invitrogen) using specific primers (*nad6* cRT 500R for the fragment downstream of the cleavage site and *nad6* 202R for the fragment upstream of it). PCR was performed with the same reverse primer (or a nested primer, *nad6* 149R) and a specific forward primer (*nad6* 517F and *nad6* 500R or *nad6* 244F and *nad6* 202R or *nad6* 149R). PCR products were cloned into pGEM-T Easy and sequenced. A similar reverse transcription was performed to check the ends of the *rps3-rpl16* transcript using the primers *rpl16*-cRT-M4 and *rps3-*cRT-R2. Primer sequences are presented in Supplementary Table 1.

### Protein electrophoresis and western blotting

BN-PAGE, NADH oxidase activity staining and western blotting were performed as previously described^16^. The blots were probed using antibodies against Nad9^44^ (1:50,000 dilution), Nad6^45^ (AS15 2926 from Agrisera at 1:1000 dilution), NDUFS4^46^ (1:5000 dilution), and CA2^47^ (1:2500 dilution) from complex I, the Rieske iron–sulfur protein of complex III^48^ (RISP, 1:5000 dilution), Cox2 of complex IV (AS04 053A from Agrisera at 1:5000 dilution), and NDB2 of the external NADH dehydrogenase^49^ (1:5000 dilution). The antibodies against AtpA of the ATP synthase (1:1000 dilution), the AOX^50^ (1:1000 dilution), and the mitochondrial membrane Porin (1:5000 dilution) were provided by Dr T. Elthon (School of Biological Sciences, University of Nebraska).

## Electronic supplementary material

Supplementary information

## Data Availability

The datasets generated in this study have been deposited in the NCBI SRA database as accession SRP158968 (accession numbers SRR7760264–SRR7760269 for the sRNA-seq data and SRR7760270–SRR7760277 for the RNA-seq data).
