# Supplementary material for: Targeted cleavage of nad6 mRNA induced by a modified pentatricopeptide repeat protein in plant mitochondria
Source: Commun Biol. 2018 Oct 11;1:166. doi: 10.1038/s42003-018-0166-8 (PMC6181959; doi:10.1038/s42003-018-0166-8)
Supplement: Supplementary file 1 — Supplementary information [file 42003_2018_166_MOESM1_ESM.pdf]

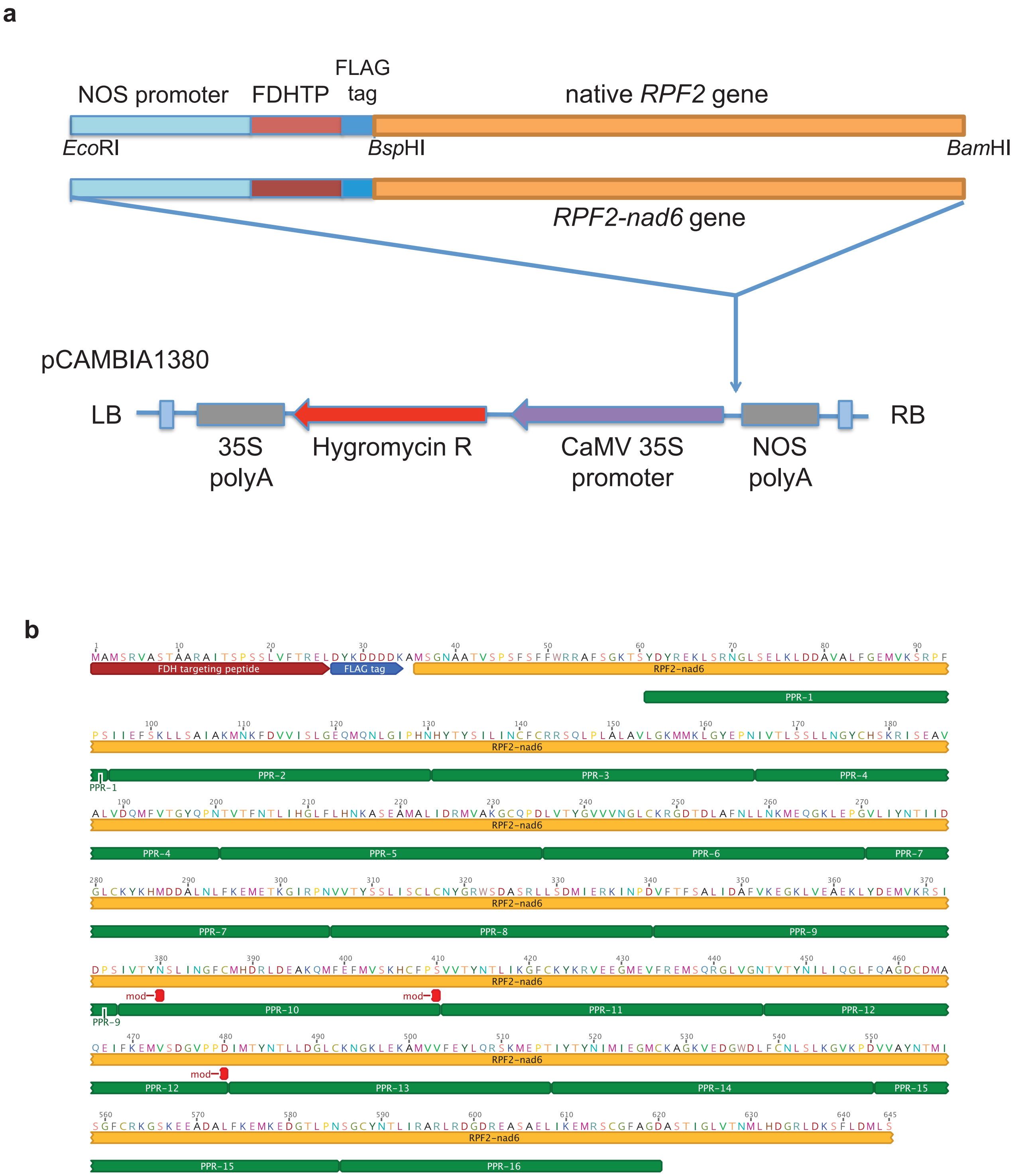

Supplementary Figure 1. **Synthetic constructs used for plant transformation.** **a**) Native RPF2 or RPF2-*nad6* gene expression was driven by the nopaline synthase promoter (light blue). The proteins were designed to be targeted to mitochondria by the formate dehydrogenase targeting peptide (FDHTP), and, following cleavage of this targeting signal, to carry a FLAG tag at the N-terminus. **b**) Annotated sequence of the RPF2-*nad6* protein. The FDHTP is coloured in brown, the FLAG tag in blue, the PPR motifs in green, and the three residues modified from native RPF2 are marked in red. The diagram was prepared in Geneious 9.1.5 [1].

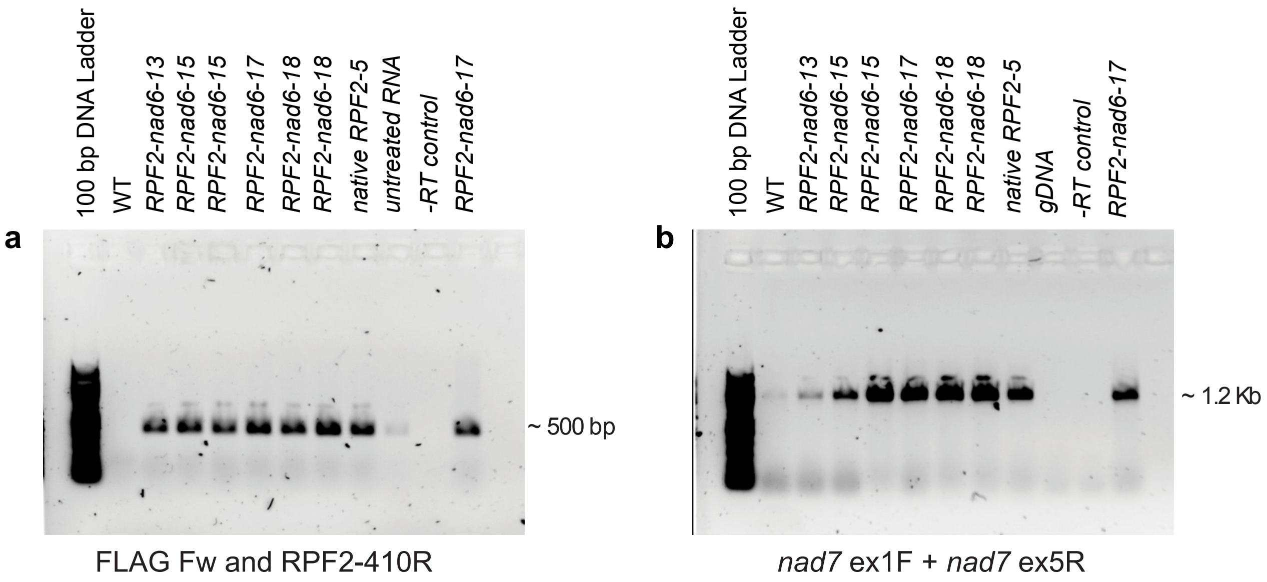

Supplementary Figure 2: RT-PCR verifying expression of the constructs in transformed plants. a) Primers FLAG Fw and RPF2-seq 410R were used to amplify a transgene-specific part of the tagged RPF2 sequence from cDNA. “Untreated RNA” diluted 1/100 used as a template shows amplification from the genomic DNA contamination in an RNA sample before DNase treatment. “-RT control” is a cDNA synthesis reaction where the reverse transcriptase was omitted, showing the effectiveness of the DNase treatment performed before the cDNA synthesis. Lines with the same names are derived from independent RNA samples from the same transgenic line. **b**) As a positive control, the expression of the endogenous *nad7* transcript was also checked in these plants (primers *nad7* ex1F and *nad7* ex5R).

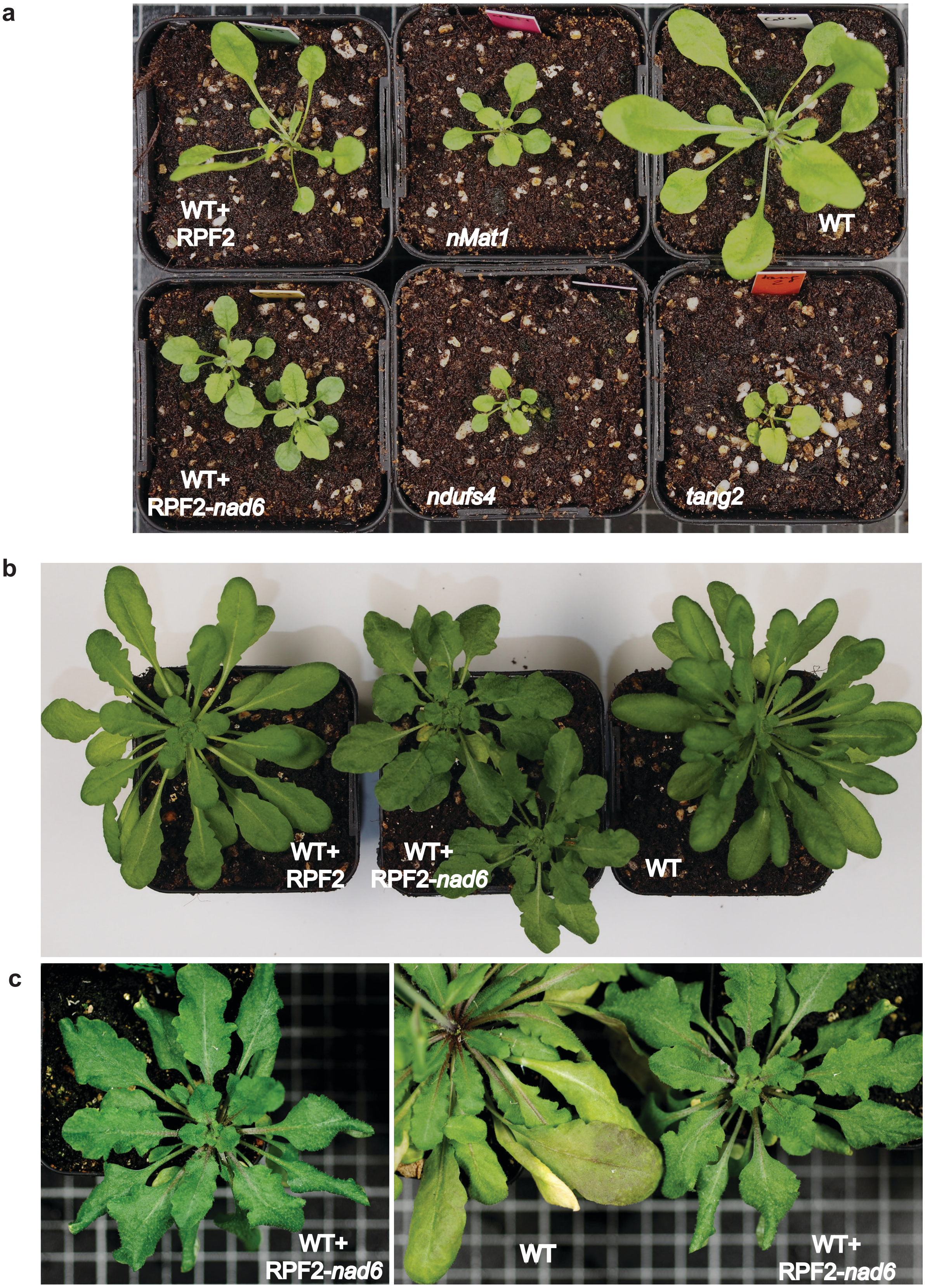

Supplementary Figure 3. Phenotypes of plants transformed with native RPF2 or RPF2-*nad6*. a) at 4 weeks next to mutants directly (*ndufs4* [2]) or indirectly (*nMat1* [3] and *tang2* [4]) impaired in complex I assembly, grown under a 16 hour photoperiod. **b**) at 8 weeks next to WT or **c**) 11 week-old plants grown under an 8 hour photoperiod.

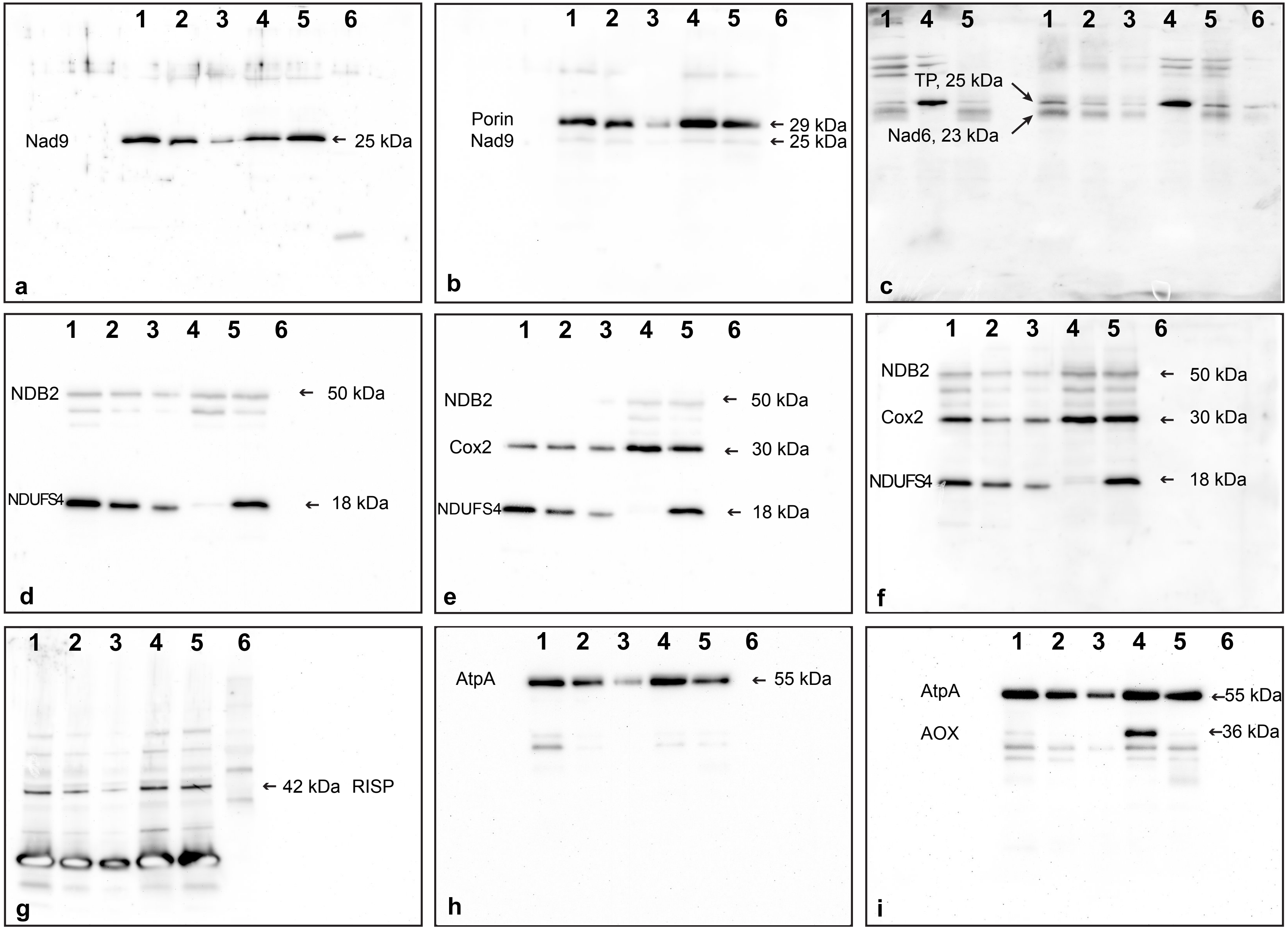

Supplementary Figure 4. Uncropped western blot membranes used in Figure 2. The names of the target proteins and their expected sizes are indicated on each membrane. Membranes shown in **a**, **b** were sequentially probed with antibodies recognising Nad9 and Porin. The membranes shown in **d**, **e**, **f** were sequentially probed with antibodies recognising NDB2, NDUFS4, Cox2 and again NDB2. The membranes shown in **h**, **i** were sequentially probed with antibodies recognising AtpA and AOX.The signal labelled TP in **c** represents a contaminating thylakoid protein which is recognised by the antibody. Lanes are 1: Col-0 100 %, 2: Col-0 50 %, 3: Col-0 25 %, 4: RPF2-*nad6*, 5: native RPF2, 6: molecular weight markers.

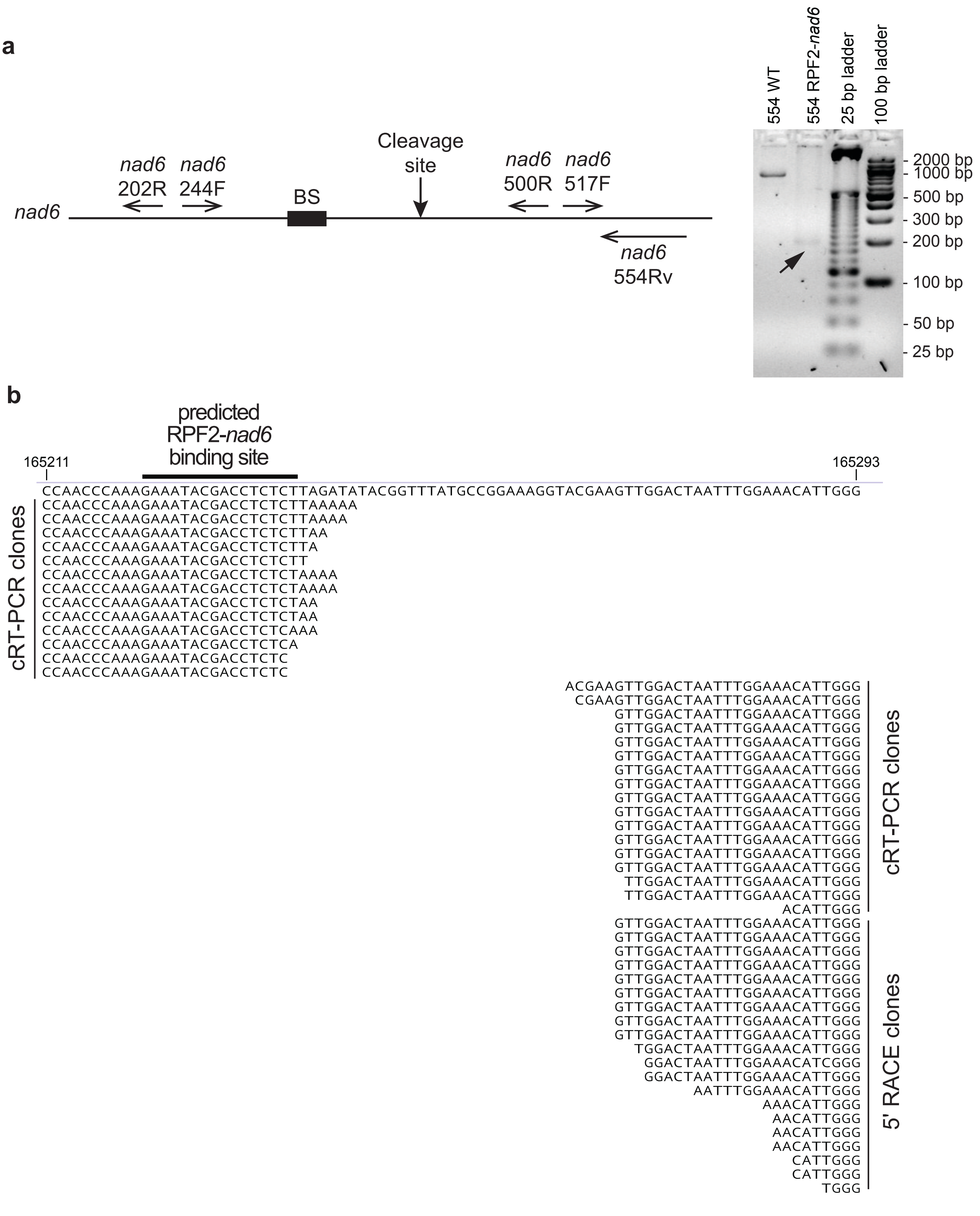

Supplementary Figure 5. Sizes and sequences of the 5'-RACE and cRT-PCR products. a) The positions of the primers used for the 5'-RACE and cRT-PCR experiments. Right panel: separation of various 5'-RACE products on a 3% low melting point agarose gel. **b**) Alignment of cRT-PCR clones obtained with the 244F and 202R primers, cRT-PCR clones obtained with the 517F and 500R primers and 5'-RACE clones obtained with the 554R primer.

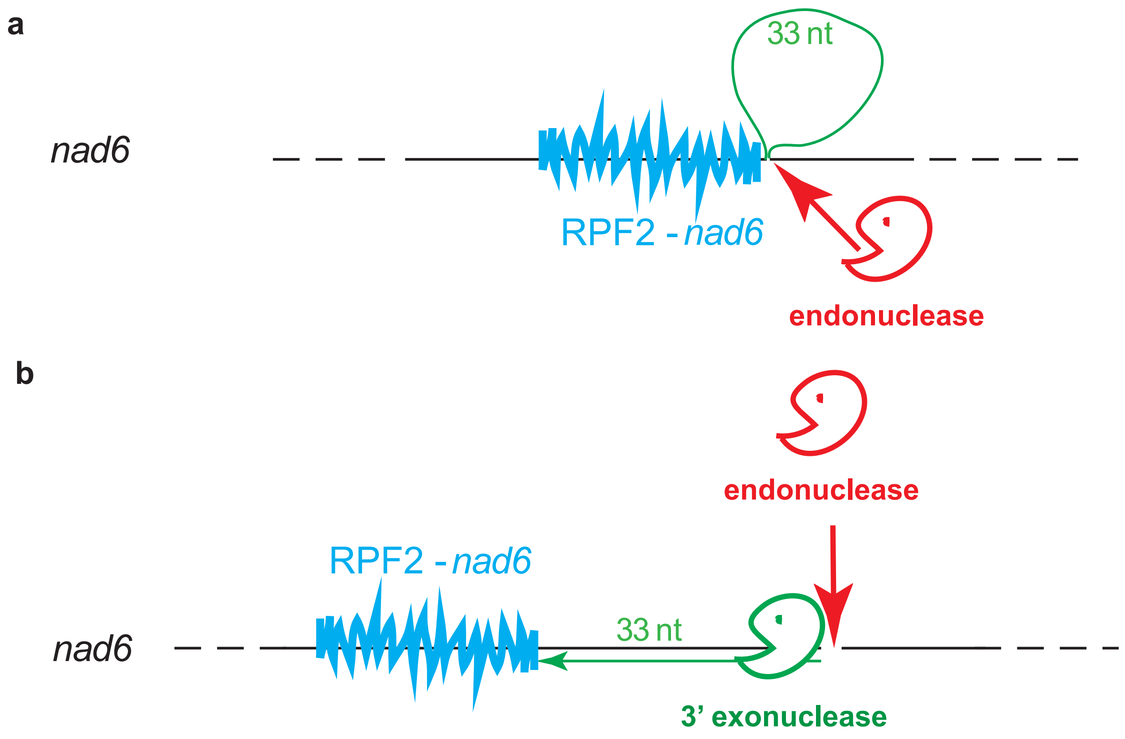

Supplementary Figure 6. Possible mechanisms for the RPF2-*nad6*-mediated cleavage of the *nad6* transcript. a) double endonucleolytic cleavage; **b**) single cleavage followed by exonucleolytic trimming.

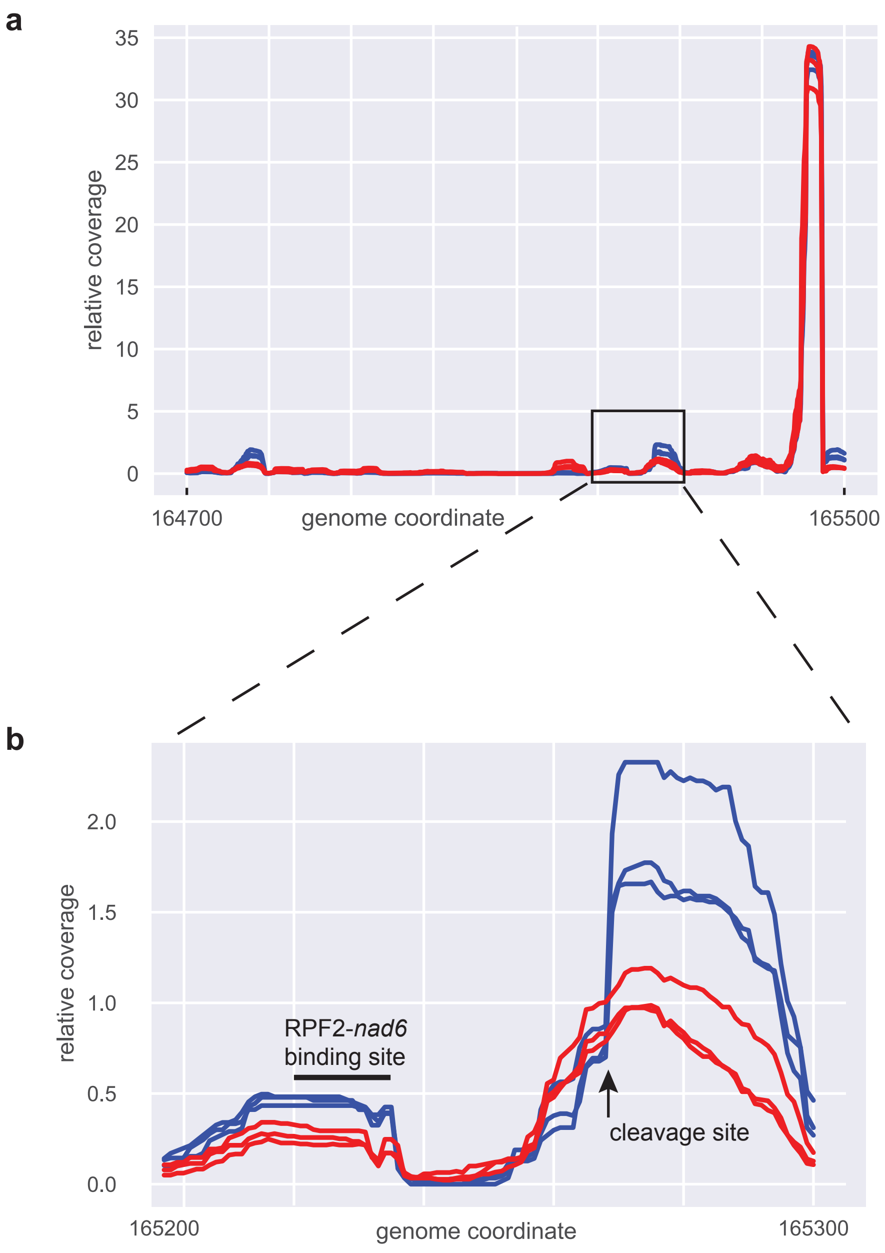

Supplementary Figure 7. Relative coverage of small RNA fragments mapping to *nad6*. Small RNA reads were mapped to the mitochondrial genome and the read coverage calculated for each base. Coverage was roughly normalised for the *nad6* region by dividing coverage at each base by the total coverage across the region, to facilitate comparison of the coverage pattern between wild-type and RPF2-*nad6* plants despite the 4-fold difference in *nad6* transcript levels. Coverage in RPF2-*nad6* is in blue, WT in red. **a**) Small RNA coverage is dominated by a prominent footprint at the 3' terminus of the *nad6* transcript [5]. **b**) Zooming in on the region of the predicted RPF2-*nad6* binding site shows that there is no support for a ~33 nt cleavage product between the RPF2-*nad6* binding site and the mapped cleavage site. The difference in coverage for small RNAs starting at the the cleavage site confirm the 5'-RACE and cRT-PCR results in Supplementary Figure 3 that are summarised in Figure 5.

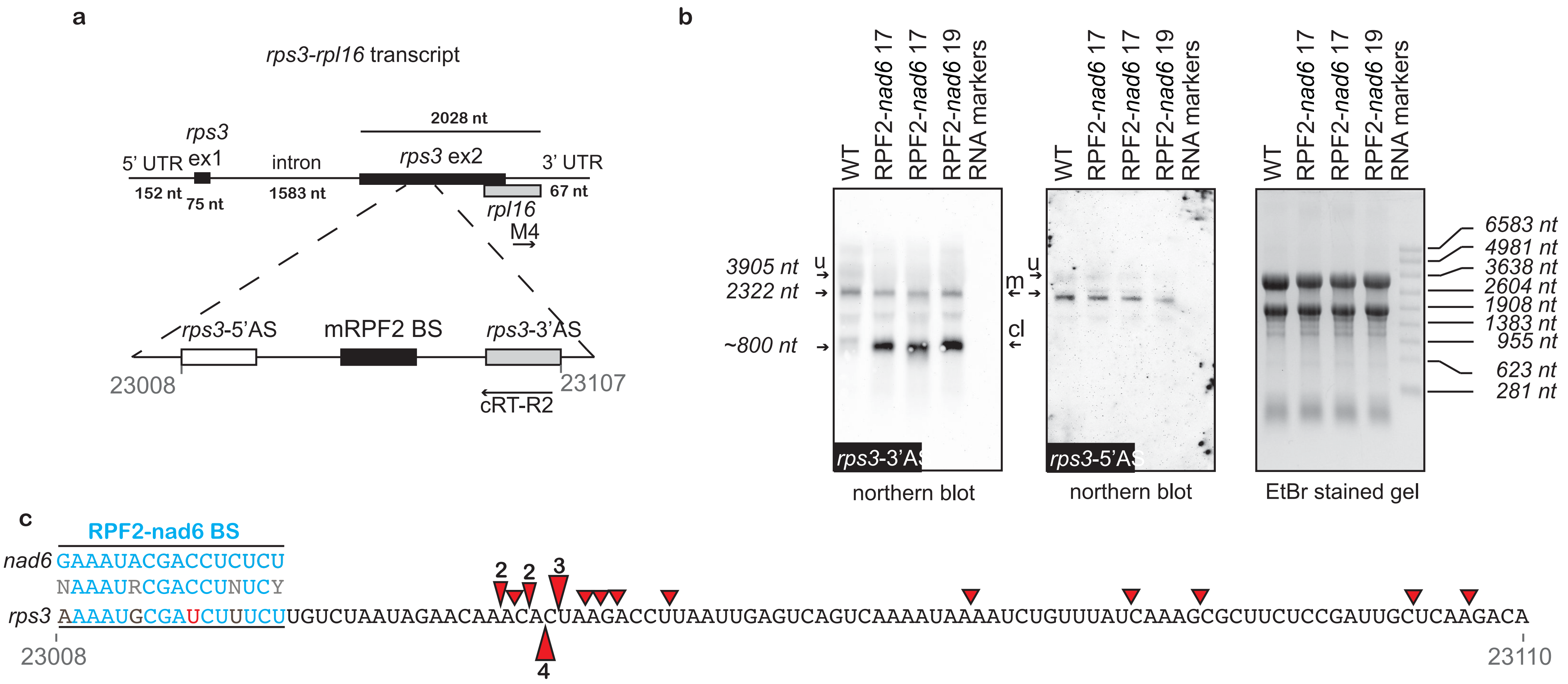

Supplementary Figure 8. Off-target cleavage of the *rps3-rpl16* transcript in RPF2-*nad6* plants. a) Schematic diagram of the *rps3-rpl16* transcript unit showing the sizes of the different regions, the site predicted to be bound by RPF2-*nad6*, and the positions bound by probes *rps3*-5' AS, *rps3*-3' AS and the primers for cRT PCR. **b**) Nothern blot of flower RNA from RPF2-*nad6* and WT plants. The probe *rps3*-3' AS hybridizes 186 nt downstream of a predicted binding site for RPF2-*nad6*. The probe *rps3*-5' AS hybridizes 474 nt upstream of the binding site. The right panel shows a gel stained with ethidium bromide prior to transfer and indicates the sizes of the hybridising RNAs (u: unspliced; m: mature; cl: cleavage product) and RNA size markers. **c**) Positions of the 5' ends of the cleaved transcripts in RPF2-*nad6* plants as mapped by cRT-PCR. The coordinates of this region on the Col-0 mitochondrial genome are 23008 to 23110**.** The predicted RPF2**-*nad6*** binding site (23008-23023) is highlighted and aligned with the *nad6* binding site (BS) and the degenerate sequence used to search for off-target sites. Grey indicates ambiguity in the search pattern. Brown indicates mismatches between the *rps3* and *nad6* sequences at positions that vary in the natural targets of RPF2. Red indicates the C to U mismatch to the search pattern at a position aligning with one of the motifs engineered to recognise the *nad6* site. The red triangles indicate the 5' ends of the cleaved transcripts as determined by cRT-PCR with primer cRT-R2. The figures near the triangles indicate the numbers of clones obtained.

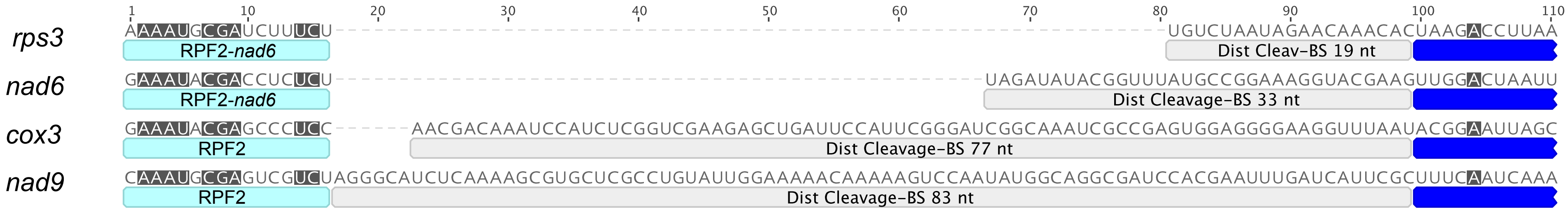

Supplementary Figure 9. Alignment of the regions comprising RPF2 binding sites and associated cleavage sites. The binding sites (BS) and the cleavage sites of the 4 targets *rps3*, *nad6*, *cox3* and *nad9* are aligned. The consensus sequences are highlighted. The alignment was performed in Geneious 9.1.5 [1].

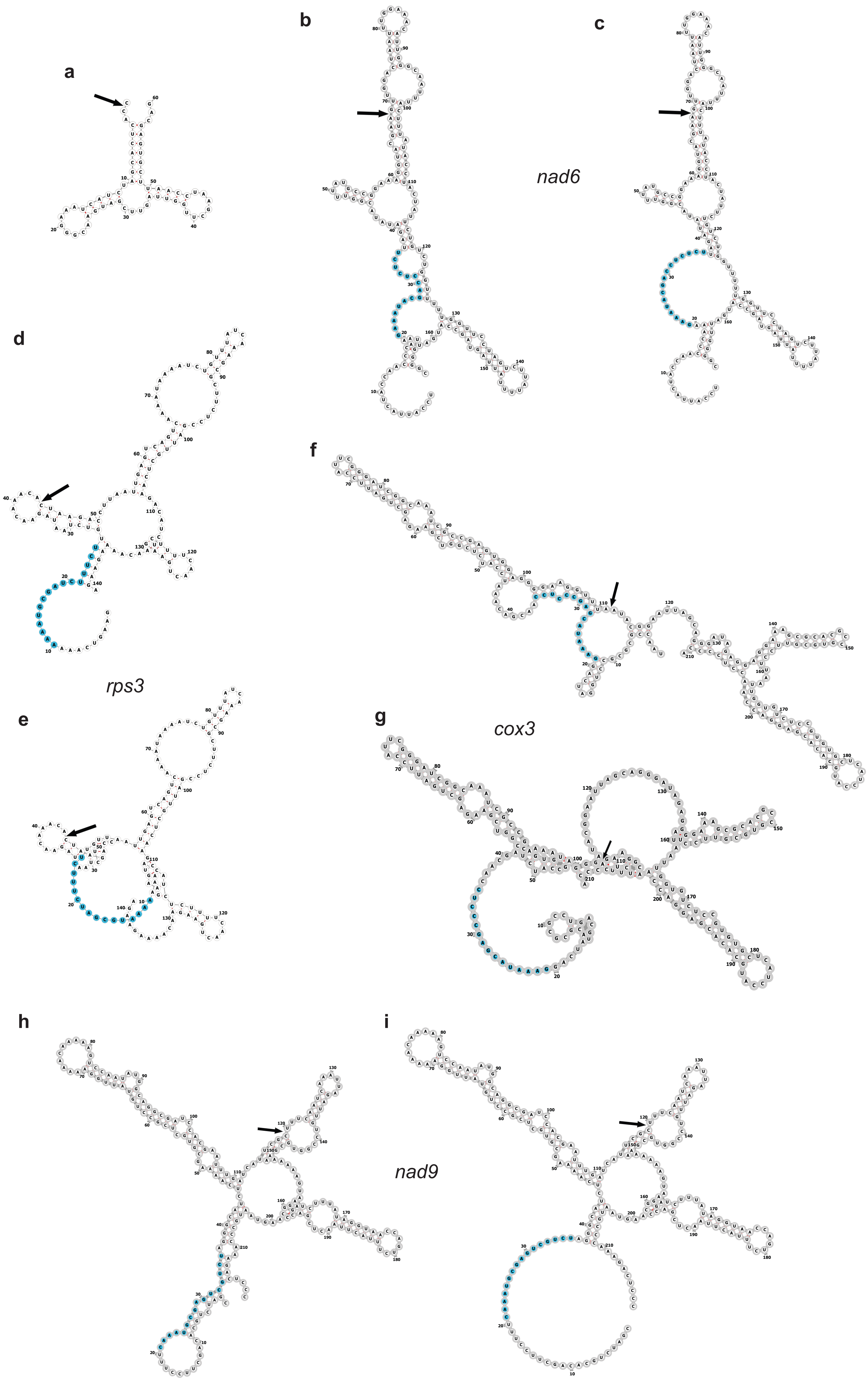

Supplementary Figure 10. RNA folding predictions around observed cleavage sites.
a) Cleavage at the 5' end of the t-element marking the 3' extremity of the *nad6* transcript in WT 6; **b, c)** cleavage site in the vicinity of the RPF2-*nad6* binding site in *nad6*; **d, e)** cleavage site in the vicinity of the RPF2-*nad6* binding site in *rps3*; **f, g)** cleavage site in the vicinity of the RPF2 binding site in *cox3*; **h, i)** cleavage site in the vicinity of the RPF2 binding site in *nad9*. In **b**, **d**, **f** and **h**, no constraints were placed on nucleotide pairing. In **c**, **e**, **g** and **i**, no pairing of the RPF2 binding site was permitted in order to simulate folding of the RNA with the protein attached. Predicted RPF2 and RPF2-*nad6* binding sites are highlighted in cyan. The cleavage sites are indicated by arrows. Folding predictions were made using the RNAfold software of the ViennaRNA Package [6].

a

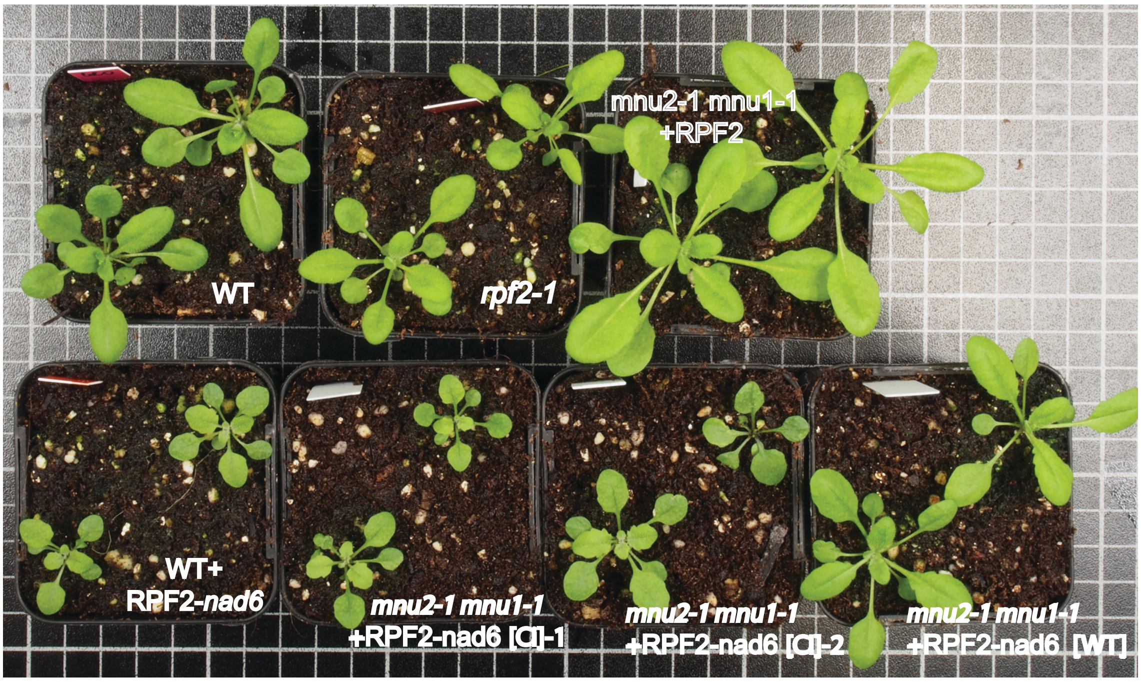

b

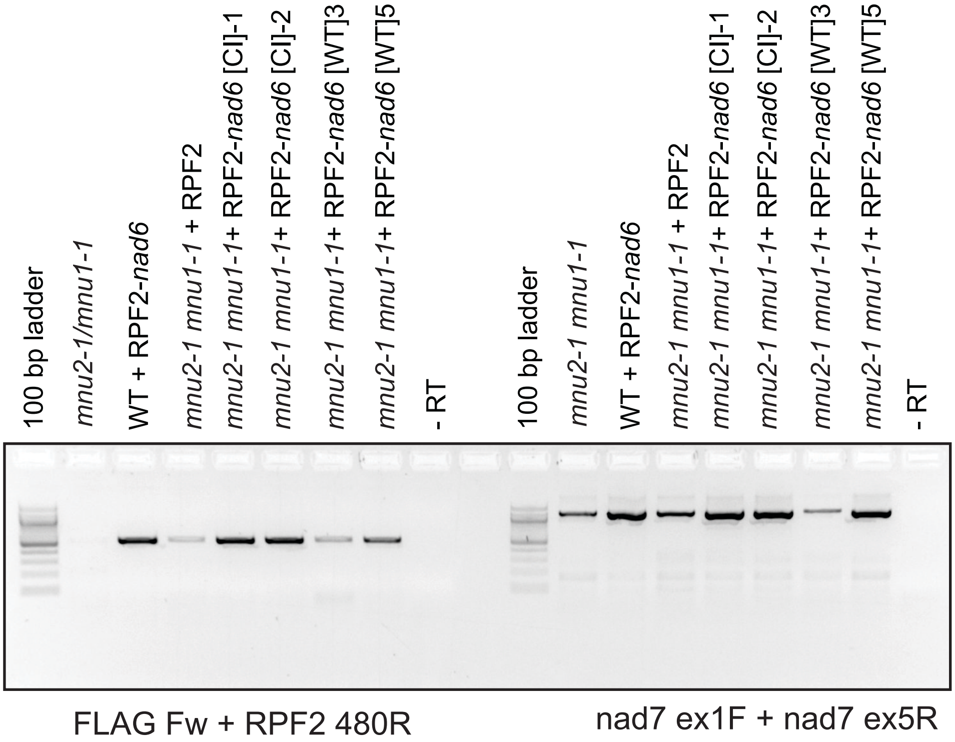

Supplementary Figure 11. Phenotypes of *mnu* plants transformed with the RPF2-*nad6* construct. a) Double mutant *mnu2-1 mnu1-1* plants [7] transformed with the RPF2 and RPF2-*nad6* constructs (T2) compared with WT and WT+ RPF2-*nad6*. [CI] indicates an apparent Complex I-deficient phenotype, [WT] indicates wild-type phenotype. **b**) RT-PCR showing the expression of the constructs in transformed plants. Primers: FLAG Fw and RPF2-420R, nad7 ex1F and nad7 ex5R. "-RT" is a cDNA synthesis reaction where the reverse transcriptase has been omitted.

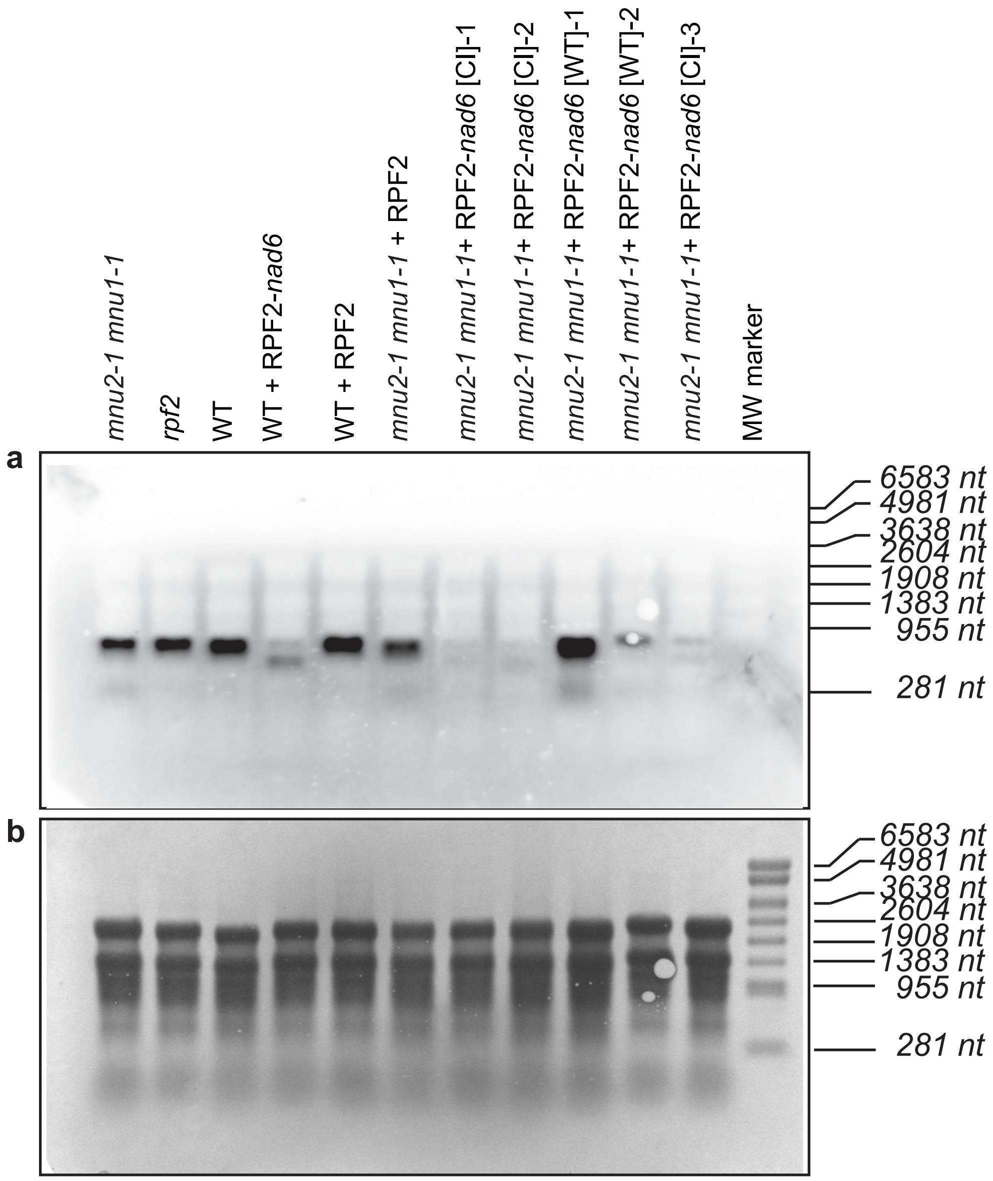

Supplementary Figure 12. RPF2-*nad6* induces cleavage of *nad6* RNA in an *mnu* mutant background. a) Leaf RNA samples were hybridized with a biotinylated probe corresponding to the RPF2-*nad6* binding site. **b)** The same membrane stained with methylene blue as a loading control. The phenotype of the plants is indicated as either wild-type [WT] or slow growing, similar to complex I mutants [CI].

Supplementary Table 1. Primers used in this work.

**Primers for cloning**	
RPF2pET*Rca* F	CAGGGCGTCATGAGCGGTAATGCTGCAACTGTTTCT
RPF2pET*Bam* H1R	TCCTGAGGATCCTTAAGAAAGCATATCGAGGAA
**Primers for RT-PCR**	
FLAG Fw	GACTACAAAGACGATGACGACAAA
RPF2-410R	AGCGAAGAAAGCGTGACAAT
nad7 ex1F	ACCTCAACATCCTGCTGCTC
nad7 ex5R	AGGTGCTTCAACTGCGGTAT
**Primers for RACE PCR**	
nad6 -554Rv	CGTCGGAATACATCCTGTCTTTTCACCTTAGTAGTCCTATGC
**Primers for circular RT PCR**	
nad6 500R	ATAGCCCCAATCATGGCTACTAAT
nad6 202R	CGAAAAGGAATGAAACGGCTA
nad6 149R	GCGAAGAAGTCGAGACCTAACA
nad6 517F	GGACTACTAAGGTGAAAAGACAGGA
nad6 244F	GAAGTATTGCGCTATTTACCAGTG
rpl16 -cRT-M4	ACGGGTTGGATTGCTCGTGT
rps3 -cRT-R2	CATATACGGATTCCCTCCACCC
**Biotinylated primers for northern blots**	
nad6 -556As	TTCGTCGGAATACATCCTGTCT
nad6 -364As	GAGAGGTCGTATTTCTTTGGGT
rps3 -3'As	AACATATACGGATTCCCTCCAC
rps3 -5'As	CGGTGCCACGAAATGATTGAGA
**Genotyping primers**	
At5g09840.A	AAGACTGTGGTATGGTTCACCTG
At5g09840.B	CTGAAACGAATCAGGCTATACCC
At5g64710.A	ACAGGTTGTTCCTCTGGGAG
At5g64710-komplR	CGTTTTGGTCAATTACCACGG

Supplementary Methods

RT-PCR

Total RNA was isolated from leaves of T2 plants using the RNeasy kit (Qiagen) and treated with Turbo-DNase (Ambion). Reverse transcription was carried out 50 min at 50ºC (after an initial step of 5 min at 25ºC) using 2-3 micrograms of total RNA (denatured 5 min at 65ºC) in a final volume of 20 µl, 1 µl random hexamers, 1 µl RNaseOUT (Invitrogen) and 1 µl Superscript III (Invitrogen) as previously described [8]. The PCR step was performed on a 1/10 dilution of the cDNA. The list of primers used is given in Supplementary Table 1.

Small RNAseq

Total RNA was isolated from 50 mg of flower buds with PureZol (BioRad). Small RNA was size selected (17-50 nt) on a denaturing acrylamide-urea gel. The libraries were prepared with the NEB Next Small RNA Library Prep Set for Illumina as described in [9] and run on an Illumina HiSeq 1500 (TrueSeq v3 kit from Illumina). After de-multiplexing of the reads, Illumina adaptor sequences were removed using bbduk (ktrim=r k=23 mink=11 hdist=1 tpe tbo) [10]. The trimmed reads were mapped to an edited version of the Col0 mitochondrial genome – NCBI accession JF729201 with known RNA editing sites modified to Y (forward strand) or R (reverse strand). Mapping was done with bbmap (semiperfectmode=t) [10]. Strand-specific genome coverage of the mapped reads was calculated with bedtools2 genomecov [11].

Supplementary References

1. Kearse, M. *et al.* Geneious Basic: an integrated and extendable desktop software platform for the organization and analysis of sequence data. *Bioinformatics* **28,** 1647–1649 (2012).
2. Meyer, E. H. *et al.* Remodeled respiration in *ndufs4* with low phosphorylation efficiency suppresses *Arabidopsis* germination and growth and alters control of metabolism at night.

Plant Physiol. **151,** 603–619 (2009).

3. Keren, I. *et al.* nMAT1, a nuclear-encoded maturase involved in the trans-splicing of *nad1* intron 1, is essential for mitochondrial complex I assembly and function. *Plant J.* **71,** 413–426 (2012).
4. Colas des Francs-Small, C. *et al.* The pentatricopeptide repeat proteins TANG2 and ORGANELLE TRANSCRIPT PROCESSING439 are involved in the splicing of the multipartite *nad5* transcript encoding a subunit of mitochondrial complex I. *Plant Physiol.* **165,** 1409–1416 (2014).
5. Forner, J., Weber, B., Thuss, S., Wildum, S. & Binder, S. Mapping of mitochondrial mRNA termini in *Arabidopsis thaliana*: t-elements contribute to 5’ and 3' end formation. *Nucleic Acids Res.* **35,** 3676–3692 (2007).
6. Lorenz, R. *et al.* ViennaRNA Package 2.0. *Algorithms Mol. Biol.* **6,** 26 (2011).
7. Stoll, B. & Binder, S. Two NYN domain containing putative nucleases are involved in transcript maturation in Arabidopsis mitochondria. *Plant J.* **85,** 278–288 (2016).
8. Falcon de Longevialle, A. *et al.* The pentatricopeptide repeat gene OTP43 is required for trans-splicing of the mitochondrial *nad1* intron 1 in *Arabidopsis thaliana*. *Plant Cell* **19,** 3256–3265 (2007).
9. Wu, W. *et al.* SOT1, a pentatricopeptide repeat protein with a small MutS-related domain, is required for correct processing of plastid 23S-4.5S rRNA precursors in *Arabidopsis thaliana*. *Plant J.* **85,** 607–621 (2016).
10. Bushnell, B. *BBMap*. sourceforge.net/projects/bbmap/
11. Quinlan, A. R. & Hall, I. M. BEDTools: a flexible suite of utilities for comparing genomic features. *Bioinformatics* **26,** 841–842 (2010).
